# Use of ctDNA in early breast cancer: analytical validity and clinical potential

**DOI:** 10.1038/s41523-024-00653-3

**Published:** 2024-06-19

**Authors:** François Panet, Andri Papakonstantinou, Maria Borrell, Joan Vivancos, Ana Vivancos, Mafalda Oliveira

**Affiliations:** 1 Breast Cancer Group, Vall d’Hebron Institute of Oncology (VHIO), Barcelona, Spain; 2 Lady Davis Institute, Jewish General Hospital, Montréal, QC Canada; 3 Department of Oncology-Pathology, Karolinska Institute, Stockholm, Sweden; 4 Department of Breast, Endocrine Tumors and Sarcomas, Karolinska Comprehensive Cancer Center, Karolinska University Hospital, Stockholm, Sweden; 5 Medical Oncology Department, Vall d’Hebron Hospital, Barcelona, Spain; 6 Cancer Genomics Group, Vall d´Hebron Institute of Oncology (VHIO), Barcelona, Spain

**Keywords:** Breast cancer, Diagnostic markers, Predictive markers, Prognostic markers

## Abstract

Circulating free tumor DNA (ctDNA) analysis is gaining popularity in precision oncology, particularly in metastatic breast cancer, as it provides non-invasive, real-time tumor information to complement tissue biopsies, allowing for tailored treatment strategies and improved patient selection in clinical trials. Its use in early breast cancer has been limited so far, due to the relatively low sensitivity of available techniques in a setting characterized by lower levels of ctDNA shedding. However, advances in sequencing and bioinformatics, as well as the use of methylome profiles, have led to an increasing interest in the application of ctDNA analysis in early breast cancer, from screening to curative treatment evaluation and minimal residual disease (MRD) detection. With multiple prospective clinical trials in this setting, ctDNA evaluation may become useful in clinical practice. This article reviews the data regarding the analytical validity of the currently available tests for ctDNA detection and the clinical potential of ctDNA analysis in early breast cancer.

## Introduction

Circulating tumor DNA (ctDNA) analysis is increasingly used in precision oncology. Tumoral DNA enters the circulation via multiple ways, including apoptosis or necrosis of tumor cells, and is a fraction of the total cell-free DNA in the bloodstream^1^. Due to DNases in circulation, renal clearance, and uptake from the liver or spleen, ctDNA has a short half-life from 16 min to 2.5 h^2,3^ and hence is a source of real-time tumor information accessible via a simple blood draw^2,4^.

Analysis of ctDNA can be used in metastatic breast cancer (BC) to identify important targets, such as mutations in the catalytic subunit alpha of phosphatidylinositol-4,5-bisphosphate 3-kinase (*PIK3CA*) or the estrogen receptor 1 (*ESR1*) gene, predictive of response to alpelisib^5,6^, or elacestrant^7,8^, respectively. Moreover, ctDNA analysis may be used to detect molecular alterations that emerge as resistance mechanisms to targeted agents^9^. In the PADA-1 trial, for instance, switching endocrine treatment to fulvestrant (with palbociclib) in patients with luminal BC with emergent *ESR1* mutation detected by ctDNA (in the absence of radiological progression), increased progression-free survival (PFS) compared to maintaining the letrozole treatment backbone^10^.

**Fig. 1:**
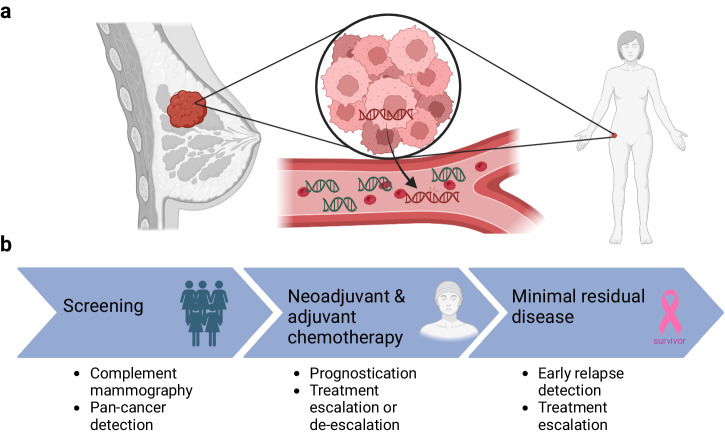
Use of ctDNA in early breast cancer. Potential and/or current applications of ctDNA in early breast cancer. **a** Breast cancer, the primary tumor or metastatic disease (radiologically visible or not), releases circulating tumor DNA (ctDNA) into the bloodstream. The amount of ctDNA is a fraction of cell-free DNA, the total DNA concentration in the blood. **b** Potential and/or current use of ctDNA throughout early breast cancer treatment journey. Created with BioRender.com.

ctDNA analysis in early BC is technically more challenging, given the relatively low sensitivity of most available tests, which is mainly related to the lower ctDNA concentration present in early BC when comparing with metastatic BC^11,12^ or other early-stage solid tumors^13^. In addition, there are also differences in the rate of ctDNA detection in early disease according to the subtype of BC, with human epidermal growth factor receptor 2 (HER2) positive and triple-negative breast cancer (TNBC) having higher levels of shed ctDNA compared to luminal BC^14,15^. Nevertheless, with advances in sequencing and bioinformatics, the use of ctDNA in early BC remains an area of interest, with numerous potential applications for enhanced treatment personalization (Fig. 1)^16,17^.

This article reviews the data regarding the clinical potential of ctDNA analysis in early BC and the analytical validity of the currently available methods.

## Methodology

**Fig. 2:**
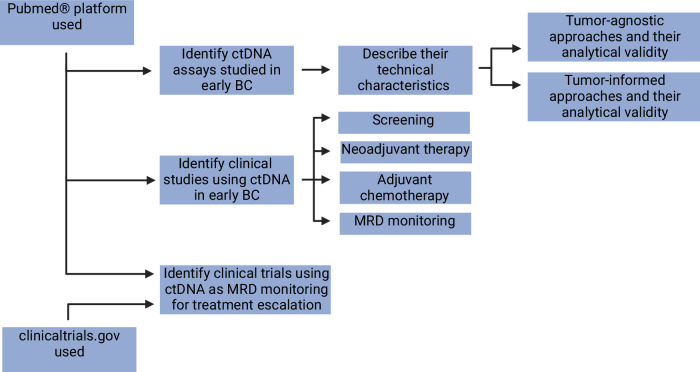
Flow diagram of the research strategy. Flow diagram of the research strategy used for the literature review. Created with BioRender.com.

Figure 2 depicts the flow diagram of the research strategy. The Pubmed® platform was used to (1) Search for the available assays that assess ctDNA in early BC, their technical characteristics, and analytical validity, and (2) Identify clinical studies or trials using ctDNA in early BC (from May 2015 to December 2023). Studies were selected for their clinical relevance and classified according to their use in different settings: screening, during neoadjuvant therapy (NAT) (Table 1), in the adjuvant setting (Table 2), and monitoring for minimal residual disease (MRD) after curative treatment to predict relapse (Table 3). In addition, clinical trials using ctDNA MRD monitoring for treatment escalation were identified from the clinicaltrials.gov database and are summarized in Table 4.

**Table 1:** Summary of clinical studies analyzing ctDNA during neoadjuvant chemotherapy (NAT)

Reference (author and year)	Number of patients (*N*) and subtypes of BC	Technique for ctDNA analysis	Median follow-up	*N* (%) of ctDNA-positivity at baseline	Relevant outcomes associated with ctDNA during NAT
Cavallone et al.^54^	*N* = 26 TNBC	Tumor-informed Pre-amplification and ddPCR	55 months	25 (96)	cDNA Pre-surgery timepoint • The average tumor VAF was lower in pCR patients, and ctDNA-positivity was a predictor of decreased RFS and OS
Li S. et al.^44^	*N* = 44 All subtypes	Tumor-agnostic Hybridization-based NGS 1021 genes	46 months	21 (48)	ctDNA during NAT • Decrease in tumor VAF: associated with the response at surgery
Rothé et al.^62^	*N* = 69 HER2+	Tumor-informed ddPCR *PIK3CA* or *TP53* mutation	Not given	28 (41)	ctDNA-positivity at baseline • Lower pCR
Lin P.H. et al.^110^	*N* = 95 All subtypes	Tumor-agnostic Amplicon-based NGS	61 months	60 (63)	ctDNA-positivity after NAT • Decreased RFS
Riva et al.^111^	*N* = 38 TNBC	Tumor-informed ddPCR for *TP53*	24 months	27 (75)	ctDNA-positivity after 1 C NAT • Lower RFS and OS
McDonald et al.^12^	*N* = 33 All subtypes	Tumor-informed TARDIS	Not given	32 (100)	Timepoint pre-surgery • Tumoral VAF 5.7 fold lower in pCR patients compared to RD
Takahashi et al.^39^	*N* = 87 All subtypes	Tumor-informed OS-MSP of *RASSF1A*	23 months	20 (23)	Timepoint pre-surgery • Tumoral VAF decreased in patients with lower RCB scores at surgery
Ortolan et al.^112^	*N* = 26 TNBC	Tumor-informed ddPCR	24 months	10/13 (77)	ctDNA-positivity pre-surgery • Worse 2-year EFS
Zhou Q. et al.^61^	*N* = 145 HR+ and TNBC	Tumor-informed Personalized amplicon-based NGS via SiMSen-Seq	Not given	63 (43)	ctDNA-positivity mid-NAT • Higher RCB score
Magbanua et al.^14^	*N* = 283 HR+ and TNBC	Tumor-informed Signatera^TM^	50 months	224 (80)	ctDNA-positivity pre-surgery • Lower DRFS for patients with RD at the surgery
Ciriaco et al.^63^	*N* = 20 HER2+ and TNBC	Tumor-informed Personalized amplicon-based NGS via Sysmex SafeSEQ	Not given	19 (95)	Timepoint pre-surgery • ctDNA-negative 93.3% accurate to predict pCR
Moss et al.^34^	*N* = 34 All subtypes	Tumor-agnostic Amplification of breast-specific methylation loci and targeted methylation analysis	Not given	22/30 (73)	Level of breast cfDNA pre-surgery • Predict the presence of RD at surgery
Cailleux et al.^113^	*N* = 44 All subtypes	Tumor-informed Signatera^TM^	36 months	22/38 (58)	ctDNA-positivity pre-surgery • Lower EFS
Parsons et al.^64^	*N* = 38 TNBC and ER low (≤ 5%)	Tumor-informed MAESTRO	Not given	38/38 (100)	Baseline to 2nd cycle of NAT • Mean decrease in tumoral VAF higher in responder

*BC* breast cancer, *cfDNA* cell-free DNA, *CI* confidence interval, *cm* centimeter, *ctDNA* circulating tumor DNA, *ddPCR* digital drop polymerase chain reaction, *DRFS* distant relapse-free survival, *EFS* event-free survival, *ER* estrogen receptor-positive, *FU* follow-up, *HER2* human epidermal growth factor receptor 2, *HR* hazard ratio, *HR* *+* hormone receptor-positive, *HR−* hormone receptor-negative, *LN* lymph node, *mid-NAT* at the middle of neoadjuvant chemotherapy, N number of patients, *NAT* neoadjuvant chemotherapy, *NGS* next-generation sequencing, *pCR* pathological complete response, *PET-CT* positron emission tomography with computed tomography, *RCB* residual cancer burden at surgery, *RD* residual disease, *RFS* relapse-free survival, *T* timepoint, *TILs* tumor-infiltrating lymphocytes, *TNBC* triple-negative breast cancer, *VAF* variant allele frequency, *WES* whole-exome sequencing, *Y* year.

**Table 2:** Summary of clinical studies analyzing ctDNA in the adjuvant setting after curative treatment

Reference (author and year)	Number of patients (*N*) and key inclusion criteria	Timepoint(s) of ctDNA analysis	Technique for ctDNA analysis	Median follow-up	Selected outcomes associated with ctDNA detection in the adjuvant setting
Stecklein et al.^46^	*N* = 80 TNBC with RD post-NAT	1 timepoint • 1–6 months after curative treatment	Tumor-agnostic amplicon-based NGS of 275 genes related to cancer	31 months	Outcomes associated with ctDNA-positivity • Lower 3 y EFS: 48% in ctDNA-positive vs 82% in ctDNA-negative • Lower 3 y OS: 50% in ctDNA-positive vs 86% in ctDNA-negative
Chen Y.-H. et al.^114^	*N* = 38 TNBC with significant RD after NAT	4 timepoints • 4 timepoints during adjuvant therapy (36 weeks duration)	Tumor-informed Amplicon-based targeted NGS. Only mutation(s) present(s) in the primary tumor were considered ctDNA-positive	24 months	Detect recurrence • Specificity 100% • Sensitivity 31% Outcomes associated with ctDNA-positivity • Lower DFS
Radovitch et al.^87^	*N* = 142 TNBC with RD post-NAT	1 timepoint • At trial entry (after surgery and radiation therapy)	Tumor-agnostic FoundationACT^TM^ or FoundationOneLiquid^TM^	17.2 months	Detect recurrence • Sensitivity 79% Outcomes associated with ctDNA-positivity • Lower DFS probability at 24 months: 50% in ctDNA-positive vs 76% in ctDNA-negative • Lower OS probability at 24 months in ctDNA-positive patients 57% vs 80% in ctDNA-negative

*CI* confidence interval, *cm* centimeter, *ctDNA* circulating tumor DNA, *DFS* disease-free survival, *EFS* event-free survival, *HR* hazard ratio, *N* number of patients, *NAT* neoadjuvant chemotherapy, *NGS* next-generation sequencing, *OS* overall survival, *RCB* residual cancer burden at surgery, *RD* residual disease, *T* timepoint, *TNBC* triple-negative breast cancer.

**Table 3:** Summary of clinical studies analyzing ctDNA for MRD monitoring (ctDNA was analyzed retrospectively in those studies)

Reference (author and year)	Number of patients (*N*) and inclusion	Frequency of ctDNA analysis	Technique for ctDNA analysis	Median follow-up	Performance of ctDNA MRD monitoring
Shaw et al.^57^	*N* = 156 Completion of curative treatment in the previous 3 years and high risk of relapse (≥ 65% at 10 years)	Every 6 months for a total of 12 years	Tumor-informed Signatera^TM^	Not reported	Detect recurrence • Specificity 95% • Sensitivity 88% The median lead time of ctDNA-positivity before clinical relapse • 10.5 months Association with ctDNA-positivity • HR for RFS 47.5 • HR for OS 84.15
Lipsyc-Sharf et al.^58^	*N* = 83 High-risk HR + HER2- more than 5 years after curative treatment	Every 6 to 12 months	Tumor-informed RaDaR^TM^	2.0 years from the first sample	Detect recurrence • Sensitivity 85.7% • Specificity 97.4% The median lead time of ctDNA-positivity before clinical relapse • 12.4 months
La Rocca,^115^	*N* = 33 TNBC Stage II and more	Every 6 months (less than 50% of patients were followed)	Tumor-informed ddPCR	5.1 years	Detect recurrence • Sensitivity 75% for distant disease in patients tested according to protocol • Specificity not reported
Garcia-Murillas et al.^116^	*N* = 144 All subtypes receiving NAT	Every 3 months for the first year, then every 6 months for a total of 5 years	Tumor-informed ddPCR	36.3 months	Detect recurrence • Sensitivity 79% • Specificity not reported The median lead time of ctDNA-positivity before clinical relapse 10.7 months Association with ctDNA-positivity • HR for RFS 17.4
Janni et al.^93^	*N* = 38 No information on subtypes	At 12 or 36 months post-diagnosis and/or at clinical relapse	Tumor-agnostic GuardantReveal^TM^	Not reported	Detect recurrence • Sensitivity 85% of distant and 15% of local relapses • Specificity 100%
Janni et al.^94^	*N* = 311 All subtypes	One timepoint at two years after the completion of adjuvant chemotherapy	Tumor-agnostic GuardantReveal^TM^	65 months	Detect recurrence • Sensitivity 34% • Specificity 97.7% The median lead time of ctDNA-positivity before clinical relapse • 7.9 months Association with ctDNA-positivity • HR for RFS 11
Elliott et al.^95^	*N* = 83 HER2- BC	Post-operative and every 3–6 months during follow-up	Tumor-agnostic Methylation analysis from GuardantINFINITY^TM^	3.7 years	ctDNA detected post-operative and/or during follow-up • *P* = 0.00021 for association with clinical recurrence
Loi et al.^92^	*N* = 178 High-risk luminal BC	At trial entry and at 24 months	Tumor-informed Signatera^TM^	Unknown	10/178 (5.6%) patients tested positive at baseline • 3 cleared ctDNA at 24 months: no relapse • 7 did not clear ctDNA at 24 months: all relapsed At 24 months 42/178 (23.6%) tested positive: all relapsed • Sensitivity for relapse 60% • Specificity for relapse 100%

*BC* breast cancer, *CI* confidence interval, *ctDNA* circulating tumor DNA, *ddPCR* digital drop polymerase chain reaction, *EFS* event-free survival, *ER* estrogen receptor-positive, *FU* follow-up, *HER2* human epidermal growth factor receptor 2, *HR* hazard ratio, *HR* *+* hormone receptor-positive, *HR−* hormone receptor-negative, *N* number of patients, *NAT* neoadjuvant chemotherapy, *NGS* next-generation sequencing, *OS* overall survival, P *P* value, *RD* residual disease, *RFS* relapse-free survival, *TNBC* triple-negative breast cancer, *VAF* variant allele frequency, *WES* whole-exome sequencing.

**Table 4:** Prospective interventional clinical trials using ctDNA MRD monitoring randomizing patients to escalate treatment after curative surgery

Clinical trial identification number and name	Key inclusion and patients enrolled or planned	ctDNA assay used and frequency of ctDNA analysis during the screening period	Treatment escalation in ctDNA-positive patients without metastatic disease on imaging	Key outcomes	Status and results published (if present)
NCT03145961, c-TRAK TN^89^	*N* = 161 enrolled High-risk resected TNBC	Tumor-informed ddPCR	Randomization 2:1 to pembrolizumab or observation (protocol amendment closed the observation group)	Co-primary endpoints • ctDNA detection rate • Sustained ctDNA clearance rate on pembrolizumab	Completed Detect recurrence • Specificity 99.8% • Sensitivity not reported. 7 patients relapsed without ctDNA detection • High proportion of ctDNA-positive patients had metastatic disease on imaging • High rate of treatment refusal • None cleared ctDNA on pembrolizumab
NCT04915755, ZEST trial^98,117^	*N* = 800 (planned) Stage I–III HR + /HER2- gBRCA or TNBC after curative treatment	Tumor-informed Signatera^TM^ Frequency of ctDNA analysis is different depending on the time elapsed since surgery	Randomization 1:1 to niraparib or placebo (in two cohorts depending if gBRCA or wtBRCA)	Primary outcomes related to treatment tolerance	Terminated by the sponsor • Higher than expected ctDNA-positive patients had metastatic disease on imaging
NCT04567420, DARE^118,119^	*N* = up to 1000 (expected) Stage II-III High-risk ER+ ( ≥ 10%) /HER2- on standard adjuvant ET	Tumor-informed Signatera^TM^ Testing during routine clinical visits (recommended every 4–6 months)	Randomization to palbociclib-fulvestrant or standard adjuvant endocrine therapy	Co-primary endpoints • Incidence of ctDNA detection • Effect of Palbociclib plus fulvestrant on RFS	Recruiting, • Update (2023) N = 542 enrolled screening period • 37 ctDNA-positive • 10 with metastatic disease on imaging • 22 randomized
NCT03285412, LEADER part II^120,121^	*N* = 120 (expected) T1c-T4c, any N, ER+ ( ≥ 10%) /HER2- on adjuvant ET	Tumor-informed Signatera^TM^ No information on the timing of ctDNA testing	Randomization to ribociclib and ET or standard adjuvant ET	• Rate of ctDNA clearance • DFS	Recruiting • Update (2023) N = 191 enrolled screening period • 17 ctDNA-positive • 4 with metastatic disease on imaging • 12 randomized
NCT04985266, TRAK-ER^122^	*N* = 1100 (expected) High-risk ER+ ( ≥ 10%) /HER2- on standard adjuvant endocrine therapy	Tumor-informed RaDaR^TM^ Every 3 months for up to 3 years	Randomization to palbociclib-fulvestrant or standard adjuvant ET	• Incidence of positive ctDNA result during surveillance • Effect of Palbociclib plus fulvestrant on RFS	Recruiting
NCT05512364, TREAT-ctDNA Elacestrant^123^	*N* = 220 (expected) High-risk ER+ ( ≥ 10%) /HER2- on standard adjuvant endocrine therapy	Tumor-informed RaDaR^TM^ ctDNA tested at multiple timepoints	Randomization to elacestrant or standard adjuvant ET	• Effect of elacestrant on DMFS • ctDNA elimination rate at month 1	Recruiting

*ctDNA* circulating tumor DNA, *ddPCR* digital drop polymerase chain reaction, *DFS* disease-free survival, *DMFS* distant metastasis free survival, *ER* *+* estrogen receptor-positive, *ET* endocrine therapy, *gBRCA* germline pathogenic *BRCA* mutation, *HER2* human epidermal growth factor receptor 2, *N* number of patients, *NGS* next-generation sequencing, *RFS* relapse-free survival, *TNBC* triple-negative breast cancer, *VAF* variant allele frequency, *wtBRCA* absence of germline pathogenic BRCA mutation.

## Technical aspects of ctDNA detection in early breast cancer

**Fig. 3:**
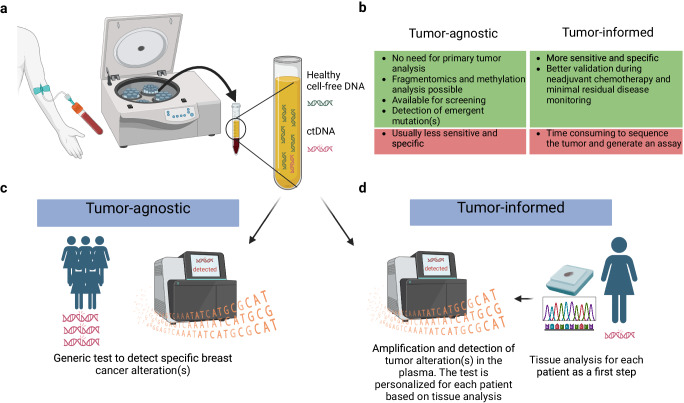
Schematic representation of tumor-agnostic and tumor-informed circulating tumor DNA (ctDNA) analysis. Schematic representation of tumor-agnostic and tumor-informed circulating tumor DNA (ctDNA) analysis. **a** ctDNA analysis involves centrifuging the blood sample to isolate plasma, followed by DNA extraction (DNA extraction not shown). **b** Advantages and disadvantages of tumor-agnostic and tumor-informed assays. **c** Tumor-agnostic assay utilizes a standardized test for each individual without requiring prior knowledge of the tumor’s genetic information. It is worth noting that different types of tests exist, but each platform performs the same analysis on patients. **d** Tumor-informed ctDNA analysis consists of two steps. First, the primary tumor is analyzed, usually through sequencing, to identify specific tumor alterations. Specific primers or probes are made for these mutations. The second step is to analyze the DNA from the plasma for the presence of tumor-specific mutations. Created with BioRender.com.

Analysis of ctDNA is conducted on DNA extracted from plasma, a product of the centrifugation of whole blood (Fig. 3a)^17^. Sequencing and bioinformatic methods for ctDNA analysis are constantly evolving, and multiple platforms are available. These platforms can be broadly divided into: (1) tumor-agnostic and (2) tumor-informed methods (Fig. 3a–d)^18^.

Tumor-agnostic approaches do not require prior knowledge of existing tumor mutations^19^, and consequently the same assay of a given platform is used for every patient. In contrast, tumor-informed approaches require the sequencing of the tumor tissue and the subsequent development of a specific ctDNA assay unique to each patient. Both methods have advantages and limitations (Fig. 3b). For instance, while the latter is generally more sensitive for detecting low tumoral variant allele frequencies (VAF)^18^, it is also more time-consuming because it requires prior sequencing of the tumor and the development of a personalized assay for every patient as a first step^14^. In addition, tumor-informed methods cannot identify emerging mutations not present in the sequencing of the tissue biopsy nor can it be used for BC screening given the lack of known tumor mutation status at this stage^19^.

The term “analytical validity” refers to the ability with which a particular genetic characteristic is identified in a given laboratory test^20^. Though there is no universally standardized approach to conclusively determine the analytical validity of a specific test, it must take into account several key aspects, such as: (1) the determination of its limit of detection (LoD), which is the lowest tumoral VAF an assay can reliably detect at a certain confidence interval; (2) its clinical sensitivity and specificity, that informs about false negative and false-positive rates; and (3) its robustness^21^. Most ctDNA assays currently used for research purposes in early BC have incompletely reported their analytical validity (see below), but development strategies of such assays are generally focused on increasing their sensitivity, given the low tumor fraction that is normally present in early BC^22^.

In the next paragraphs, the different methods for ctDNA detection and analysis will be described, together with the existing evidence regarding their analytical validity whenever available.

## Tumor-agnostic approaches

### Next-generation sequencing (NGS) panels for genomic variants

NGS is a versatile method used for high-throughput sequencing of DNA fragments. In DNA applications, sequencing reads derived from the sample libraries are aligned to a reference genome, allowing to infer the original sequence or quantify the relative signal of particular genomic regions. In oncology, these methods are frequently used for mutation, gene fusion, and copy number alterations detection^23^. Briefly, an NGS sequencing test can be described by its depth and breadth^23^. The depth refers to the level of unique reads that align to the reference genome^23^. Low-depth NGS is also referred to as “shallow”, while high-depth is often named “deep sequencing”. The breadth is the extent of genomic coverage achieved by the assay^23^. The maximum breadth is whole-genome sequencing (WGS)^23^. For any given variant, NGS analysis provides a percentage of altered or mutant sequence reads, divided by the overall coverage or total read count at that particular locus, reported as the VAF^23^. In this review, the term “tumoral VAF” will be used to indicate the tumoral fraction of total cell-free DNA. Each assay has a limit of detection (LoD), which refers to the tumoral VAF cut-off at which it can reliably identify an alteration^24^. If the tumoral VAF is lower than the LoD, this leads to a false negative result^24^.

Clonal hematopoiesis of indeterminate potential (CHIP), in turn, can be a source of false-positive results^25^. CHIPs are age-dependent acquired mutations in hematopoietic progenitor cells in the absence of dysplasia. As cell-free DNA originates mostly from hematopoietic cells, CHIP can be mistaken for tumor mutations^23^. Recent recommendations from ESMO highlight the need to perform synchronous profiling of plasma DNA (for ctDNA analysis) and white blood cell DNA in the buffy coat (for identification of mutations related to CHIP) to rule out CHIP if necessary^26^.

As the ctDNA concentration in the blood is low, most platforms use methods to increase the signal of certain regions to increase the sensitivity of ctDNA detection at low tumoral VAFs. These methods include amplicon-based and hybridization (also referred to as hybrid) capture targeted NGS.

### Amplicon-based targeted NGS

In amplicon-based NGS, unique molecular identifiers (UMIs), also named barcodes, are added to each original DNA fragment before the PCR amplification phase to tag them. These are used to suppress false-positive signals introduced by PCR. They then perform amplification of the library in a nested-like approach and use in-depth NGS to sequence the amplicon products. Methods using this chemistry include Safe Sequencing System (Safe-SeqS) and Simple, multiplexed, PCR-based barcoding of DNA for sensitive mutation detection using sequencing (SiMSen-seq) and can detect mutant allele at a frequency ranging from 0.1–0.02%^27,28^. Since these NGS assays use PCR, variations to these methods can amplify tumor-specific mutations before NGS and thus, also be applied in tumor-informed approaches (personalized amplicon-based NGS).

### Hybridization (hybrid) capture targeted NGS

Hybrid capture-based approaches, which increase the breadth of the method, utilize specific/complementary biotinylated probes to enrich the library for genomic regions of interest by isolating them from other non-targeted regions^29^. Cancer Personalized Profiling by Deep Sequencing (CAPP-Seq) was first described in lung cancer and utilized biotinylated bait oligonucleotide to enrich for ~125 kilobase (kb) followed by deep sequencing^29^. It could identify tumoral VAF down to ~0.02% at 96% specificity^29^. Guardant360^TM^ uses this approach and it was the first ctDNA assay approved by the Food and Drug Administration (FDA) for tumoral mutation profiling across all advanced solid-cancer sites^30^. Similarly, GuardantReveal™, which is used for MRD monitoring, targets a ~500 kb panel targeting both somatic and epigenomic regions using biotinylated bait oligonucleotides^31^. It then amplifies and sequences the sample via two types of analyses: one for genomic variants (single nucleotide variants, insertion-deletion alterations) and the other for methylation^31^. GuardantReveal™ excludes false-positive signals due to CHIPs via a bioinformatics pipeline without buffy coat analysis^31^.

### Methylation analysis

DNA methylation, which occurs primarily on a cytosine ring within cytosine-guanine (CpG) dinucleotides, is important to control gene expression, and its patterns are known to be the best cell-of-origin biomarker^32,33^. Each type of tissue has a unique pattern and can therefore be identified by methylation analysis^34^. Furthermore, each subtype of BC has a unique methylation pattern that can be detected in the bloodstream^35^. Commercially available whole-genome bisulfite sequencing (WGBS), a type of NGS, enables DNA methylation analysis at high breadth^13^ on cell-free DNA, enabling the detection of methylation patterns specific for BC^36^. Bisulfite sequencing involves a conversion with sodium bisulfite which changes unmethylated cytosine to uracil without affecting methylated cytosine^33^. However, there is some DNA loss during bisulfite conversion affecting sensitivity^37^.

To reduce cost and increase the depth of cover at sites of interest, targeted methylation analysis can be performed. Targeted methylation analysis using biotinylated probes on bisulfite-converted DNA is currently used in the pan-cancer screening blood test Galleri^TM^ commercialized by GRAIL^38^.

Another strategy, which also requires bisulfite conversion, is to conduct one-step methylation-specific PCR (OS-MSP) which can detect methylation at one specific locus^39^. GuardantReveal^TM^ does not use bisulfite conversion but tags and UMIs for methylation detection^40^. Similar to GuardantReveal^TM^, GuardantINFINITY^TM^ is a research ctDNA assay that combines genomic and epigenomic profiling, but its methylation analysis (without bisulfite conversion) targets 15 megabases (Mb) which is larger than GuardantReveal^TM41^. A methylation score was developed to discriminate patients with and without cancer and could identify 95% of patients with BC (*N* = 139, no information on stage)^41^. At low ctDNA concentration, which is common in early BC, methylation analysis was better than tumor-agnostic genomic profiling targeting somatic variants to quantify ctDNA or identify patients with BC^42,43^.

## Analytical validity of the different tumor-agnostic approaches

Most studies using tumor-agnostic research NGS did not report complete analytical validity of their assays but excluded some genes to control for CHIPs, a common source of false positives^44–46^. Criteria for calling positivity in tumor-agnostic tests are available in Supplementary Table S1.

### Analytical validity of the different tumor-agnostic approaches used as a pan-cancer screening test

The pan-cancer screening test Galleri^TM^ has published an analytical validity report and a computational approach to estimate the tumoral fraction of total cell-free DNA^47^. By diluting samples from six cancer patients, they estimated the tumoral fraction LoD at 95% probability to be from 0.51% to ≤0.09% (one patient with BC of undisclosed stage had a tumoral fraction LoD at 95% probability of ≤0.09%)^47^. Reproducibility testing in 20 cancer patients (81 samples) reported an agreement of 95.1%^47^. Further analysis from non-cancer patients (*N* = 583, 1204 samples) demonstrated a specificity of 99.3%^47^.

For CancerSEEK, only specificity analysis, including 812 healthy controls (>99% specificity) and bioinformatics tools, has been described^48^.

### Analytical validity of the different tumor-agnostic approaches used during neoadjuvant therapy, adjuvant therapy, and MRD monitoring

FoundationOne ACT^TM^ hybridization-capture NGS was validated using cancer cell lines mixed with healthy DNA at different concentrations^49^. The assay had a >95% sensitivity for base substitution and rearrangement at VAF 0.25–0.5%^49^. However, the detection of CNV was dependent on the degree of amplification^49^.

GuardantReveal™ was validated in colorectal cancer^50^. GuardantINFINITY^TM^ was validated using cell lines, cancer patients and healthy-control samples^43^. With an input of 5 ng of cell-free DNA of patients with BC, tumoral fraction of cell-free DNA LoD for sample-level methylation was 0.023%, which was lower than for single nucleotide variations, insertions, or deletions^43^. Moreover, the sample-level methylation analysis had a false-positive rate of 0% when analyzing the plasma of six healthy donors^43^.

## Tumor-informed approaches

### Droplet digital PCR (ddPCR)

ddPCR, similar to quantitative PCR (qPCR), involves the use of fluorescent reagents that emit particular wavelengths during amplification^51^. This fluorescence is then detected and quantified. Compared to qPCR, ddPCR increases precision and reproducibility^51^. Overall, ddPCR involves partitioning the sample of DNA separating individual molecules into multiple droplets where isolated PCR reactions will occur, and then analyzing each one independently^52^. ddPCR ctDNA assays can be tumor-agnostic if it uses the same primers amplifying a generic mutation, usually for a targetable mutation in the metastatic setting^53^. In early BC, ddPCR is used as a tumor-informed approach requiring tumor sequencing and the creation of specific PCR primers for the detection of personalized mutations^54,55^. The sensitivity depends on the primers, but it frequently enables the detection of tumoral low-frequency mutation in cell-free DNA in concentrations as low as 1:10,000 copies^56^.

### Personalized amplicon-based NGS

Newer molecular biology tools, involving a combination of processes, enable the detection of low tumoral VAF mutation in the plasma. These include RaDaR^TM^ for MRD detection by Inivata^TM^, targeted digital sequencing (TARDIS) by Exact sciences^TM^, and the Signatera^TM^ MRD detection test^12,57,58^. These methods can reliably assess ctDNA at a tumor VAF of less than 0.1% and they monitor more than 10 tumor-specific mutations by using UMIs followed by amplification via multiplex PCR and in-depth NGS^12,58,59^.

### Personalized hybridization-based NGS

Similarly to amplicon-based NGS, new assays increase the sensitivity of ctDNA detection by using tumor-specific mutation probes for hybridization and signal enrichment^60^. One example is the Minor Allele Enriched Sequencing Through Recognition Oligonucleotides (MAESTRO) assay, which uses smaller probes than typical hybridization capture and can isolate more than 10,000 specific mutations^60^. The assay involves sequencing of both tumor and healthy patient tissue to identify true tumoral mutations and can reliably assess ctDNA at a tumor VAF of less than 0.1%^60^.

**Fig. 4:**
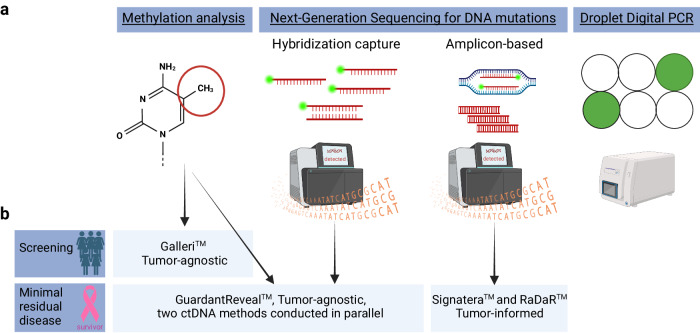
Schematic representation of circulating tumor DNA (ctDNA) analysis methods and commercially available ctDNA assays in early breast cancer. **a** Schematic representation of the different methods of ctDNA analysis. **b** Commercially available ctDNA assay in early breast cancer according to the setting where it is offered and the analysis method (arrow). To note, none of the tests are recommended by any major guidelines. Created with BioRender.com.

The different methods for ctDNA analysis and the commercially available tests in early BC are summarized in Fig. 4.

## Analytical validity of the different tumor-informed approaches

Tumor-informed assays follow specific tumoral somatic variants, however, the criteria for a positive ctDNA result are unique for each assay which usually include steps to assure specificity and/or sensitivity (Supplementary Table 2). One way to control for sensitivity is to use a commercial sample known to be positive for a somatic variant or methylation^39,61^. For example, Zhou et al. used a commercial control to determine the sensitivity of their personalized amplicon-based NGS (SiMSen-seq) in some variants (mutations followed) at multiple dilutions and established a cut-off of tumoral VAF ≥0.1% for a ctDNA sample to be classified as positive^61^.

Most tumor-informed methods used controls or algorithms at a predefined power to consider a sample as ctDNA-positive (Supplementary Table 2)^54,62–64^. For example, Cavallone et al. tested each variant followed by their ddPCR on samples from three healthy donors, and a cut-off of two standard deviations above the result of the mean of the controls was set for positivity^54^. MAESTRO uses a 90% predefined prediction power to consider a sample positive, and this assay was negative in a specificity analysis of eight plasma samples from healthy donors^60,64^. McDonald et al., in the analytical validation of TARDIS, used commercial reference samples at different expected tumoral VAF with assays following different numbers of variants^12^. When following 16 variants, a sample-level sensitivity of 87.5% at a tumoral VAF of 0.03% was observed with a specificity of 100% on unmutated control^12^.

The commercially available tumor-informed tests have been further validated. Signatera^TM^ uses a large set of negative control samples (∼1000) for background error and has a predefined algorithm threshold (0.97) for calling variants positive^65^. In analytical validity studies using three cell lines (including two BC cell lines), a commercial mutation mixture and healthy control at various dilutions, a sensitivity of 93–100% at a tumoral VAF of 0.01% and 100% at a tumoral VAF of 0.03% with a specificity of >99.8%, was observed^59^. RaDaR^TM^ was tested by controlling eight patients assays, two of whom with BC, and reported a specificity of 100% when tested on multiple samples (18 healthy donors and five cancer cell lines^66^). Moreover, dilutions from three cell lines (including two BC cell lines) were used to test sensitivity and demonstrated a 95% probability LoD at 0.0011% tumoral VAF^66^. Interestingly, tumor-informed assays including more than one variant reported increased sensitivity with an increased number of mutations followed^12,59,64,66^. Importantly, both Signatera^TM^ and RaDaR^TM^ include an analysis of the buffy coat to eliminate false positives due to CHIPs^14,66^.

In summary, there is significant variability in the methods used for assessing ctDNA analytical validity, with different bioinformatical tools, sensitivity, and specificity control or cut-off. For tumor-informed approaches, which are often used in multiple tumor sites (e.g., RaDaR^TM^, Signatera^TM^), it is unknown if they need to be validated in each specific tumor site, given they are ctDNA assays personalized for each patient. A statement from the American Society of Clinical Oncology and the College of American Pathologists Joint Review emphasizes the necessity for further validation plus standardization of diverse technologies^67^. The FDA is leading the Sequencing Quality Control Phase 2 project aiming to evaluate the use of genomic technologies in clinical applications and compared the analytical validity of some ctDNA tests but none of those were dedicated to early BC^21^. Comprehensive analytical validity assessments of ctDNA assays are essential if ctDNA is to enter routine clinical practice, as it is a fundamental factor to ascertain their real clinical utility^68^.

## Clinical use of ctDNA in early breast cancer

### Detection of ctDNA for breast cancer screening

DNA methylation pattern is organ or disease-specific and is increasingly used for cancer screening^69^. However, in contexts where robust and efficient screening strategies, as seen in breast cancer, are already in place, integrating ctDNA analysis becomes more challenging. In the next paragraphs we will summarize the available evidence of the use of ctDNA detection in BC screening and possible ways to incorporate it in future screening programs.

### Performance of ctDNA pan-cancer screening tools in BC

Jamshidi et al. tested different pan-cancer screening platforms on the plasma of healthy control or newly diagnosed cancer patients from the Circulating Cell-free Genome Atlas (CCGA): WGBS for methylation, targeted NGS for single nucleotide variation or WGS^13^. Methylation analysis was the best method for pan-cancer screening because it can detect patterns at low tumoral VAF, and there is no interference due to CHIPs^13,70^. The same team used targeted methylation analysis to develop the commercially available pan-cancer screening blood test Galleri^TM^ (from GRAIL)^71^. The test was evaluated in the validation cohort of the CCGA prospective, case–control, observational, longitudinal study (cancer: *N* = 2823; non-cancer: *N* = 1254) and reported a detection rate of 30.5% for newly diagnosed BC compared to 51.5% for all cancers while maintaining a specificity of 99.5%^71^. However, in BC, there were large discrepancies across stages and different subtypes of breast cancer. For instance, the detection rate for stage I BC was 2.6% vs 90.9% for stage IV, underscoring the challenge of detecting early-stage BC via ctDNA analysis^71^.

In the prospective interventional PATHFINDER trial, 6662 cancer-free subjects aged ≥50 years were screened with the Galleri^TM^ test and followed for one year. Ninety-two patients had a positive Galleri^TM^ test result, with 35 being true positive (14 of which stage I–II, none were BC) and 57 false-positive^72^. In this trial, five metastatic relapses of BC were diagnosed with the help of a positive Galleri^TM^ test, but 17 BC, including 13 stages I–II, were diagnosed via another method, mostly screening mammograms, again underscoring the difficulty of screening with ctDNA to diagnose early BC^72^. Large prospective interventional cohort trials are ongoing, such as the STRIVE study (NCT03085888), which aims to assess the performance of the Galleri^TM^ test for pan-cancer screening and plans to enroll 100,000 women during their mammographic screening^73^. If the sensitivity of the assay improves or an effective combination strategy with mammography is validated, methylation analysis would have the potential to become a screening tool for early BC. However, there are still controversies regarding the false-positive rate at the population level that need to be studied before such an approach can be recommended^74^.

Another pan-cancer screening test, CancerSEEK, combines ctDNA detection with proteomic analysis^48^. It screens for eight prevalent cancers, including breast cancer, and looks at an amplicon-based targeted NGS of 16 genes involved in cancer together with eight cancer-specific proteins in the blood via an immunoassay^48^. In an observational study involving 1005 patients with untreated early cancer and 812 healthy controls, median sensitivity was 70% for detection of early cancer in the eight included tumor types while maintaining a specificity over 99%^48^. However, there was a huge discrepancy in detection between tumor sites. Similar to Galleri^TM^, the detection rate for early BC was low: 33% compared to 98% detection for ovarian cancer^48^. Furthermore, in DETECT-A, a prospective interventional study using a modified version of CancerSEEK and further steps to reduce the interferences of CHIPs (*N* = 10,006 women aged 65–75 years old), almost all BC (26/27) were diagnosed using another method mostly routine screening mammogram^75^.

Although extrapolation from different studies should be avoided, it is remarkable that both pan-cancer ctDNA-based screening methods CancerSEEK and Galleri^TM^ consistently report low detection rates for early BC^48,71,72^. Detection by mammography screening in combination with an inherent low amount of shed ctDNA in early-stage BC compared with other tumor sites, probably explains the limited success of ctDNA as a screening tool^71^.

### ctDNA detection as a complementary BC screening tool

Another approach would be to perform ctDNA analysis only in those patients with abnormal mammography findings, as many patients have suspicious lesions based on screening mammograms but only a minority are diagnosed with breast cancer^76^.

ctDNA has been studied in this setting to discriminate patients with neoplasia or benign disease^77^. Barbirou et al. collected plasma samples of 32 dense-breast patients with an abnormal mammogram, 20 of whom were subsequently diagnosed with cancer^77^. They used WGS NGS ctDNA analysis and found no significant variation of the tumoral fraction of cell-free DNA between the group with cancer and those with benign disease, underscoring the low level of ctDNA in early BC^77^. However, they found specific BC mutations only in the BC group, highlighting the fact that the use of more sensitive and specific methods in this setting is essential^77^.

Aiming to increase the detection rate, a prospective trial is currently enrolling patient candidates for a breast biopsy utilizing different types of liquid biopsies plus ctDNA and a learning algorithm on breast tomosynthesis to discriminate between benign lesions and BC (NCT04781062)^78^.

### Clinical applications of ctDNA assessment in early BC

NAT is recommended for stage II-III TNBC, HER2-positive, and high-risk luminal BC. The analysis of ctDNA during NAT may have the potential to implement escalation or de-escalation strategies to better tailor treatment^79^. While appealing, this approach is currently experimental and should only be used in the context of clinical research.

In the next paragraphs, we will describe the evidence regarding the clinical validity and/or utility of ctDNA assessment in early BC. A summary of relevant studies analyzing ctDNA in patients receiving NAT is shown in Table 1. Those studies conducted ctDNA assays on banked samples evaluating the prognostic potential of ctDNA at different timepoints.

### ctDNA assessment before NAT

The sensitivity to detect ctDNA at baseline depends on the analysis technique employed, as well as the stage and subtype of breast cancer (Table 1). With a highly sensitive test like TARDIS, MacDonald et al. demonstrated that the level of tumoral VAF is more informative than the presence or absence of ctDNA^12^. In this study, 100% of patients had ctDNA detected at baseline and most patients who achieved pathological complete response (pCR) had detectable ctDNA after NAT; however, VAF in these patients was lower compared to patients with residual disease; 0.003% vs. 0.018%, respectively (*P* = 0.0057)^12^.

Using a sensitive tumor-informed ddPCR that detected ctDNA in 96% of the patients at baseline, Cavallone et al. also demonstrated that patients with more advanced staging and/or grade had a higher tumoral VAF at baseline, and the reduction in tumoral VAF after NAT was a stronger predictor of pCR compared to ctDNA detection itself^54^.

With the personalized hybridization-capture technique MAESTRO, Parsons et al. followed a median of 1000 tumor-specific mutations in 38 TNBC or ER-low (≤5%) patients receiving NAT in the TBCRC 030 trial and found that baseline detection was 100% but the mean tumoral VAF was higher in larger tumor size and patients with positive LN^64^. Moreover, the degree of mean tumoral VAF reduction was higher in responding patients during NAT (285-fold in responding vs. 24-fold in non-responding). These data also highlight the fact that if the method for ctDNA analysis is less sensitive, the absence of detection of ctDNA at baseline may represent a tumor with lower stage and/or grade, and not necessarily a non-shedding tumor^64^.

We previously published a meta-analysis showing that the detection of ctDNA at baseline was associated with worse relapse-free survival (RFS) and overall survival (OS), but could not predict the achievement of pCR^79^. One might speculate whether the correlation with RFS and OS could be due to higher ctDNA detection in higher stages, and thereof is identifying patients with intrinsic poorer prognosis^80^. The divergence of outcomes with pCR could be related to differences in ctDNA shedding in different breast cancer subtypes, as higher grade TNBC has increased ctDNA detection and pCR rates compared to luminal tumors^14,81^. The features of the ctDNA test and their analytical validity are of paramount importance while interpreting the results of these studies.

### ctDNA assessment during NAT

In patients treated with NAT, achievement of pCR at surgery, or lack thereof, is currently used as a surrogate of prognosis and as a guide for (de)escalation strategies of adjuvant therapy^82,83^. Recent studies and the meta-analysis published by the authors confirm that ctDNA adds important prognostic information and could be an additional surrogate for treatment tailoring during (neo)adjuvant therapy^14,79,84^.

Studies during NAT report a decrease in ctDNA detection^14,54^ or tumor VAF^12,64^ to be associated with improved pCR or RFS. However, the optimal timing of ctDNA evaluation in the neoadjuvant setting is still unclear. Recently, some groups have identified the timepoint after NAT and before surgery as the most important for prognostic information^14,85^. For instance, using a tumor-informed ddPCR tracking five mutations, Roseshter et al. described that among TNBC patients with residual disease after NAT this timepoint was the strongest predictor of RFS and OS (*P* < 0.0001 and *P* = 0.002, respectively)^84,85^. In a large cohort of HER2-negative BC receiving NAT, Magbanua et al. also identified ctDNA status at the timepoint after the last NAT cycle and before surgery as important^14^. Using the Signatera^TM^ tumor-informed approach, it was demonstrated that in HER2- tumors with residual disease, ctDNA-positivity before surgery was associated with worse distant recurrence-free survival (DRFS)^14^. This result was similar regardless of residual cancer burden (RCB) status, proving that ctDNA detection adds prognostic information to residual disease at surgery^14^. If these observations are confirmed, strategies for treatment de-escalation would be of interest in patients with no detectable ctDNA after NAT, given their excellent prognosis^84^. This approach might spare such patients of currently standard adjuvant treatment of arguable benefit.

The studies during curative treatment evaluation all retrospectively analyzed ctDNA. Importantly, prospective interventional trials based on ctDNA in this setting are now being planned. For example, the I-SPY 2 trial is testing the utility of ctDNA combined with MRI and biopsy for NAT treatment escalation in non-responding patients^14^ aiming at improved possibilities of NAT personalization^86^.

### ctDNA assessment during the adjuvant period (immediate post-operative)

Tumor-agnostic NGS ctDNA analysis has been studied in early TNBC after NAT and surgery (Table 2). Radovich et al. conducted a hybridization-capture NGS test (FoundationOne®) on TNBC with residual disease at the inclusion to the BRE12-158 study, evaluating adjuvant treatment consisting of molecular-guided therapy vs standard of care^87^. At trial entry, 63% of patients were ctDNA-positive with at least one mutation, copy number variation (CNV), or rearrangement^87^. DFS probability at 24 months was 50% in the ctDNA-positive group and 76% in the negative one (HR for DFS 2.67, 95% CI, 1.28–5.57; *P* = .009)^87^. The high false-positive rate seen on this test can be due to CHIPs which are frequent when certain tumor-agnostic assays are used, underscoring the need to control for this variable in this setting^25,88^.

For better prediction of recurrence, specific and sensitive assays have been conducted over multiple timepoints after surgery (Table 3, section below).

### Monitoring of MRD after curative treatment

Specific ctDNA analysis conducted over multiple timepoints can be used to detect recurrence before clinical relapse (Table 3)^57,89^. Although not yet routinely used nor recommended by any major guidelines, two tumor-informed (Signatera^TM^ and RaDaR^TM^) and one tumor-agnostic test (GuardantReveal^TM^) are commercially available in the United States for this purpose. They can detect MRD after curative treatment in multiple solid tumor sites (breast, colon, lung for all three tests, plus bladder for Signatera^TM^ and head and neck squamous cell carcinoma for RaDaR^TM^)^90,91^, although their clinical utility in BC is not yet established.

Shaw et al. reported a sensitivity of 88% and specificity of 95% with a median lead time of more than 10 months before relapse by testing with Signatera^TM^ every 6 months in high-risk patients after curative treatment for their BC^57^. In addition, in an exploratory analysis of the monarchE trial in luminal BC (*N* = 178), Signatera^TM^ was conducted at baseline and after 24 months^92^. Ten patients tested positive at baseline (10/178, 5.6%) and those who cleared their ctDNA after 24 months remained disease-free while patients with continued detection relapsed, suggesting that ctDNA may be a valuable tool to monitor adjuvant treatment in this context^92^.

In another study, Lipsyc-Sharf et al. demonstrated a sensitivity of 100% for metastatic recurrence with a median lead time of 12.4 months by testing high-risk patients after curative treatment for their luminal BC with RaDaR^TM^ every 6–12 months^58^. However, one patient with a local recurrence was not detected leading to a total sensitivity for any type of relapse of 85.7%^58^.

In an analysis of 38 patients after curative treatment for early BC of whom 20 relapsed, GuardantReveal^TM^ was positive in 14% (1/7) of local recurrence and 11/13 (85%) of metastatic relapses, but the majority of the tests were conducted at clinical recurrence^93^. In another analysis of 311 stage I–III patients after curative treatment, GuardantReveal^TM^ was conducted at only one timepoint around 2 years after adjuvant chemotherapy and detected 34% (13/38) of distant relapses^94^. Moreover, the tumor-agnostic methylation analysis of GuardantINFINITY^TM^ was studied in a cohort of 83 TNBC and luminal patients, and a positive ctDNA result after the surgery or during follow-up was strongly associated with clinical recurrence (*P* = 0.00021)^95^.

However, it is unknown whether earlier MRD detection via ctDNA improves outcome. The prospective SURVIVE study in early BC after curative treatment (NCT05658172) is currently enrolling, comparing outcomes in a standard and intensive surveillance arm, including tumor-informed ctDNA analysis via RaDaR^TM^, a circulating tumor cells test, and tumor markers^96^.

It is important to note that most of these studies are retrospective analysis of prospectively collected samples. For this reason, only the lead-in time between ctDNA detection and clinical relapse can be described, not the proportion of patients with metastatic disease at the time of ctDNA-positivity. This important question could be addressed in the prospective c-TRAK TN trial (see below).

### Clinical utility of MRD detection in interventional clinical trials

Although ctDNA tumor-informed MRD monitoring is highly sensitive to detect recurrence with a median lead time of many months, it is currently unknown if early therapeutic intervention based on ctDNA alone would significantly impact BC survival. Currently, the c-TRAK TN trial by Turner et al. is the only large prospective trial testing an intervention based on ctDNA in early BC with outcomes available^89^. This study investigated treatment escalation with pembrolizumab in patients with TNBC and residual disease post-NAT^89^. Serial tumor-informed ctDNA ddPCR analysis were performed every 3 months and if the ctDNA became positive without metastatic disease on imaging, patients were randomized 2:1 to pembrolizumab vs. observation^89^. Of the 161 patients enrolled in the screening phase, only five received pembrolizumab, and all relapsed. This low number of subjects randomized to pembrolizumab was due to a large proportion of the ctDNA-positive subgroup having overt metastatic disease on imaging; others refused to receive treatment^89^.

The c-TRAK TN trial highlights several questions moving forward. Some of them are: (1) the need for large cohorts in trials investigating interventions based on ctDNA monitoring; (2) the impact of systemic imaging after a positive ctDNA result on the accrual into the randomization phase (3) the need for sensitive screening methods for early detection of metastatic disease before radiological relapse; (4) the definition of the adequate timepoints for MRD assessment, to improve clinical feasibility. Interestingly, in c-TRAK TN two methods for ctDNA detection—ddPCR and RaDaR^TM^—were retrospectively compared in a translational exploratory analysis^97^. The latter detected ctDNA a median of 1.4 months before the ddPCR used in the trial, and 47.9% of ctDNA-positive patients would have tested positive at an earlier timepoint if RaDaR^TM^ was used instead of ddPCR^97^. One might speculate that if a positive ctDNA result had been identified earlier, more patients would have been randomized in the trial and at an earlier timepoint.

In April 2023, the ZEST trial (NCT04915755) in TNBC was terminated prematurely, due to low accrual in the treatment-escalation section as a result of a high rate of metastatic disease found on imaging when the ctDNA tested positive^89,98^. It is important to note that systemic imaging during routine follow-up after curative treatment is not standard of care. The detection of overt metastatic disease at the time of a positive ctDNA result in ZEST and c-TRAK TN, which had a clear impact on the accrual of these trials in the treatment-escalation part, raises the question of whether such an approach should be revisited, especially in the context of high-risk disease.

Several clinical interventional trials using tumor-informed ctDNA monitoring after curative therapy are currently open and their results are highly anticipated (Table 4). Three trials test the efficacy of a CDK4/6 inhibitor and endocrine therapy in ctDNA-positive patients with resected luminal BC (NCT04567420, NCT03285412, NCT04985266), while TREAT-ctDNA will use ctDNA to randomize patients to the selective estrogen receptor degrader (SERD) elacestrant or standard endocrine therapy (NCT05512364). It is interesting to note that the prospective interventional trials currently enrolling evaluating a treatment escalation with ctDNA are in luminal BC, a subtype that relapses over a longer timeframe and is associated with longer OS in the metastatic setting compared to TNBC^99^. Those different biological characteristics may render luminal BC a better candidate than TNBC for treatment escalation following a positive ctDNA test during MRD monitoring.

## Future directions

ctDNA detection in early BC is a promising tool in screening, curative treatment evaluation, and MRD monitoring. However, open questions regarding optimal patient selection, timepoints of the testing, and technical aspects of the test must be taken into consideration. Moving forward, it will be of utmost importance to know which patients to test, when to test, and which test to use along the patient journey.

One key aspect is the improvement of sensitivity and specificity of the tests, and the field is expanding. Such an example is cell-free DNA fragmentomics analysis, referring to the study of cell-free DNA fragments, focusing on their size, nucleosome positioning, and end-fragment signature^100^. These aspects were not previously considered in genomic variants or methylation analysis of ctDNA, but they contain valuable information^101^. The length of the fragments can be analyzed using current tumor-agnostic NGS assays^100^. For example, a study by Cristiano et al. utilized shallow WGS to analyze genome-wide fragmentation patterns on cell-free DNA in 208 newly diagnosed cancer patients and 215 healthy controls^102^ and could identify 57% of early BC (31 out of 54) at a specificity of 98%^102^. Notably, the sensitivity increased when they combined fragmentation patterns and targeted mutation analysis on ctDNA, which demonstrates they can be complementary^102^. Another way fragment lengths of cell-free DNA can increase ctDNA sensitivity is through signal enrichment, since fragments of ctDNA are generally shorter compared to healthy cell-free DNA, enhancing the signal of specific lengths can improve detection^103^. Using an in silico fragment lengths selection on a tumor-informed custom-capture panel ctDNA analysis, Wan et al. could detect 10 out of 16 samples of newly diagnosed stage I–II breast cancer; a sensitivity of 62.5% at a specificity of 90% (AUC = 0.81)^104^. Fragment length in silico analysis on an NGS panel is particularly useful for distinguishing between tumoral and CHIPs mutations, thus potentially improving the performance of existing tumor-agnostic assays^105,106^. Fragmentomics can be performed at a low cost by analyzing data from existing assays^107^ and could have numerous potential applications to complement ctDNA analysis, but further validation in BC is necessary^101^.

Retrospective analysis of large prospective clinical trials, such as monarchE, NATALEE, and others, may strengthen the evidence regarding the importance of MRD detection as a prognostic factor in early BC and help inform prospective clinical trials^92^. In another hand, ongoing clinical trials using ctDNA as a tool to change treatment in the early BC space will also provide important information regarding the clinical utility of this strategy and will help inform the second generation of prospective clinical trials.

Finally, ctDNA can also be found in non-blood sources and its analysis in urine and breast milk has been studied in early BC^108^. Saura et al., for instance, have demonstrated that ctDNA in breast milk had a higher sensitivity to detect post-partum breast cancer compared to plasma using a tumor-informed ddPCR assay^109^. A prospective trial is currently ongoing to evaluate the value of breast milk ctDNA screening in the post-partum period. Further improvement in ctDNA analysis and samples from other biological liquids could enhance the sensitivity of assays, particularly in early luminal BC, and could potentially be incorporated as a screening strategy in this subtype of BC.

ctDNA analysis in early BC has been limited due to the relatively low sensitivity of available techniques in a setting characterized by low levels of ctDNA shedding. However, advances in sequencing and bioinformatics, as well as the use of methylome profiles, have led to an increasing interest in the application of ctDNA analysis in this setting, from screening to curative treatment evaluation and MRD detection. A careful assessment of the analytical and clinical validity of each available ctDNA test is fundamental to correctly interpret the available results of the use of ctDNA in early BC. Moving forward, it is expected that prospective clinical trials will provide the evidence for clinical utility that is necessary to incorporate ctDNA analysis in routine practice in early BC.

### Supplementary information

Supplementary Information file: Use of ctDNA in early breast cancer: analytical validity and clinical potential
