# Supplementary material for: Use of ctDNA in early breast cancer: analytical validity and clinical potential
Source: NPJ Breast Cancer. 2024 Jun 19;10:50. doi: 10.1038/s41523-024-00653-3 (PMC11187121; doi:10.1038/s41523-024-00653-3)
Supplement: Supplementary file 1 — Supplementary Information file: Use of ctDNA in early breast cancer: analytical validity and clinical potential [file 41523_2024_653_MOESM1_ESM.pdf]

Supplementary Information file: Use of ctDNA in early breast cancer: analytical validity and clinical potential

Supplementary Table 1: Criteria for ctDNA positivity of tumor-agnostic assays

Author and ref	ctDNA assay technique	Criteria for ctDNA positivity
Schrag et al[1]	Targeted methylation analysis<br>Galleri™	• Computational algorithm
Cohen et al[2], Lennon et al[3]	Amplicon-based NGS<br>CancerSEEK	• The mutation must be in the COSMIC database or inactivate a tumor suppressor gene<br>• Statistical confirmation and control on WBC DNA to exclude CHIPs when a mutation is detected
Stecklein et al[4]	Amplicon-based NGS of 275 genes related to cancer	• Tumoral VAF ≥ 3%<br>• Patients with mutations only in *DNMT3A*, *TET2*, *ASXL1*, or *JAK2* were classified as ctDNA-negative as most likely arising from CHIPs
Radovitch et al[5,6]	Hybridization-based targeted NGS of cancer-related genes<br>FoundationACT™ or FoundationOneLiquid™	• Statistical model used for calling a ctDNA sample positive
Li S. et al[7-9]	Hybridization-based NGS of 1021 genes	• CHIPs mutation filtering, excluding mutations in *DNMT3A, IDH1,* and *IDH2* and specific alterations within *ATM, GNAS*, and *JAK2*<br>• No clear criteria for ctDNA positivity were given
Lin P.H. et al[10]	Tumor-agnostic<br>Amplicon-based NGS	• Presence of a pathogenic or likely pathogenic mutation according to the American College of Medical Genomics and Genetics (ACMG) guidelines

		• Tumor-sequencing in some case to confirm the tumoral origin of mutation
Janni et al[11,12]	Hybridization-based NGS and methylation analysis<br>GuardantReveal™	• Bioinformatics pipeline software, trained to detect the presence of ctDNA based on multiple analytic features, including genomic variation (single-nucleotide variants and insertion-deletion alterations) and epigenomic signals, and to exclude common sources of interference such as CHIPs
Elliott et al[13,14]	Tumor-agnostic<br>Methylation analysis from GuardantINFINITY™	• Bioinformatics pipeline software trained to detect cancer based on epigenomic signals

Abbreviations: ctDNA: circulating tumor DNA, CHIPs: Clonal hematopoiesis of indeterminate potential, NGS: next-generation sequencing, SNV: single nucleotide variation, VAF: Variant allele frequency WBC: white blood cell

Supplementary Table 2: Criteria for ctDNA positivity of tumor-informed assays

Author and ref	ctDNA assay technique	Number of somatic mutations followed	Criteria for ctDNA positivity
Cavallone et al[15], Roseshter et al[16], Basik et al[17]	ddPCR	5	Two standard deviations above the control (assay conducted on the plasma of three healthy donors)
Garcia-Murillas et al[18,19], Turner et al (c-TRAK TN)[20]	ddPCR	1 or 2	≥ 2 positive droplets.<br>• To confirm a positive result, it was repeated on a 2nd sample from the same timepoint in the c-TRAK TN trial
Ciriaco et al[21]	Amplicon-based NGS via Sysmex SafeSEQ	1 to 6	≥ 3 copies/mL and 3 times the value of the background established for each variant (from commercial healthy genomic DNA)

Parsons et al (TBCRC 030 trial)[22,23]	hybridization-based NGS<br>MAESTRO	319 to 1000 (Median 1000)	Predefined 90% power. If less than 10 mutations were identified, they were reviewed manually
McDonald et al[24]	amplicon-based NGS<br>TARDIS	6 to 115 (average 30)	• Minimum 2 read families (RFs)<br>• For each mutation: at least 0.5 mutant copies/reaction (mutant RFs / total RFs)<br>• If only one variant is present: at least two different RF lengths<br>Each sample-level positive ctDNA result was confirmed using the Bonferroni corrected p-value <0.05
Rothé et al[25]	ddPCR	1	Statistically higher than the control (mean of 8 assays conducted on mutation-negative cell lines or healthy donors)
Riva et al[26]	ddPCR	1	≥ 2 positive droplets<br>• Cut-off established testing their assay on a minimum of 5 control DNA for each variant. Specificity 99.4%
Zhou et al[27]	ddPCR	average 2.6 mutations	Tumoral VAF ≥ 0.1%<br>• Established testing their assay on commercial mutated DNA for some variants
Ortolan et al[28], La Rocca et al[29]	ddPCR	1	≥ 3 positive droplets in all replicates
Chen Y.-H. et al[30]	Amplicon-based targeted NGS.	Not applicable	Only mutation(s) present(s) in the primary tumor were considered ctDNA-positive
Takahashi et al[31]	OS-MSP of *RASSF1A*	1 (methylated gene)	≥ 3.3 copies/mL<br>• Established testing their assay on commercial fully methylated DNA
Magbanua et al[32], Cailleux et al[33], Shaw et al[34],	Signatera™	16	• ≥ 2 variants present with a confidence score above a predefined algorithm threshold (0.97)

			• Exclude mutations from CHIPs (from sequencing the buffy coat and bioinformatic pipeline)
Lipsyc-Sharf et al[35]	RaDaR™	Up to 48	• A statistical model is used to assess the statistical significance of the observed mutant counts for each variant to consider a sample positive or negative<br>• Exclude mutations from CHIPs (from sequencing the buffy coat and bioinformatic pipeline)

Abbreviations: 2nd: second, CHIPs: Clonal hematopoiesis of indeterminate potential, ctDNA: circulating tumor DNA, ddPCR: digital drop polymerase chain reaction, mL: milliliter, NGS: next-generation sequencing, RF: read family, VAF: Variant allele frequency

References

1 Schrag, D. *et al.* Blood-based tests for multicancer early detection (PATHFINDER): a prospective cohort study. *The Lancet* **402**, 1251-1260 (2023).

2 Cohen, J. D. *et al.* Detection and localization of surgically resectable cancers with a multi-analyte blood test. *Science* **359**, 926-930 (2018).

3 Lennon, A. M. *et al.* Feasibility of blood testing combined with PET-CT to screen for cancer and guide intervention. *Science* **369**, eabb9601 (2020).

4 Stecklein, S. R. *et al.* ctDNA and residual cancer burden are prognostic in triple-negative breast cancer patients with residual disease. *NPJ Breast Cancer* **9**, 10 (2023).

5 Radovich, M. *et al.* Association of circulating tumor DNA and circulating tumor cells after neoadjuvant chemotherapy with disease recurrence in patients with triple-negative breast cancer: preplanned secondary analysis of the BRE12-158 randomized clinical trial. *JAMA oncology* **6**, 1410-1415 (2020).

6 Clark, T. A. *et al.* Analytical validation of a hybrid capture–based next-generation sequencing clinical assay for genomic profiling of cell-free circulating tumor DNA. *The Journal of Molecular Diagnostics* **20**, 686-702 (2018).

7 Li, S. *et al.* Circulating tumor DNA predicts the response and prognosis in patients with early breast cancer receiving neoadjuvant chemotherapy. *JCO Precision Oncology* **4**, 244-257 (2020).

8 Hu, Z.-Y. *et al.* Identifying circulating tumor DNA mutation profiles in metastatic breast cancer patients with multiline resistance. *EBioMedicine* **32**, 111-118 (2018).

9 Yang, X. *et al.* Technical validation of a next-generation sequencing assay for detecting clinically relevant levels of breast cancer–related single-nucleotide variants and copy number variants using simulated cell-free DNA. *The Journal of Molecular Diagnostics* **19**, 525-536 (2017).

10 Lin, P.-H. *et al.* Circulating tumor DNA as a predictive marker of recurrence for patients with stage II-III breast cancer treated with neoadjuvant therapy. *Frontiers in Oncology* **11**, 736769 (2021).

11 Janni, W. *et al.* Multiomic, plasma-only circulating tumor DNA (ctDNA) assay identifies breast cancer patients with minimal residual disease (MRD) and predicts distant recurrence. *Cancer Research* **82**, 3403-3403 (2022).

12 Janni, W. *et al.* PS06-06: Analysis of ctDNA for the detection of minimal residual disease (MRD) using a tissue-free, multiomic assay in patients with early-stage breast cancer. *San Antonio Breast Cancer Symposium 2023, San Antonio, USA* (2023).

13 Elliott, M. *et al.* 310P Longitudinal evaluation of circulating tumour DNA in early breast cancer using a plasma-only methylation-based assay. *Annals of Oncology* **34**, S308 (2023).

14 Greenwald, W. W. Y. *et al.* Accurate epigenomic estimates of circulating tumor fraction in large-scale clinical data. *Lung* **276**, 203 (2022).

15 Cavallone, L. *et al.* Prognostic and predictive value of circulating tumor DNA during neoadjuvant chemotherapy for triple negative breast cancer. *Scientific Reports* **10**, 1-13 (2020).

16 Roseshter, T. *et al.* Abstract P2-11-26: The prognostic role of circulating tumor DNA after neoadjuvant chemotherapy in triple negative breast cancer with residual tumor. *Cancer Research* **83**, P2-11-26-P12-11-26 (2023).

17 Basik, M. *et al.* Circulating tumor DNA after neoadjuvant chemotherapy is a better prognostic test than Residual Cancer Burden in patients with triple negative breast cancer and residual tumor. *San Antonio Breast Cancer Symposium, San Antonio, USA* (2023).

18 Garcia-Murillas, I. *et al.* Mutation tracking in circulating tumor DNA predicts relapse in early breast cancer. *Science translational medicine* **7**, 302ra133-302ra133 (2015).

19 Garcia-Murillas, I. *et al.* Assessment of molecular relapse detection in early-stage breast cancer. *JAMA oncology* **5**, 1473-1478 (2019).

20 Turner, N. C. *et al.* Results of the c-TRAK TN trial: a clinical trial utilising ctDNA mutation tracking to detect molecular residual disease and trigger intervention in patients with moderate and high-risk early stage triple negative breast cancer. *Annals of Oncology* (2022).

21 Ciriaco, N. *et al.* Clearance of ctDNA in triple-negative and HER2-positive breast cancer patients during neoadjuvant treatment is correlated with pathologic complete response. *Therapeutic Advances in Medical Oncology* **14**, 17588359221139601 (2022).

22 Parsons, H. A. *et al.* Circulating tumor DNA association with residual cancer burden after neoadjuvant chemotherapy in triple-negative breast cancer in TBCRC 030. *medRxiv*, 2023.2003. 2006.23286772 (2023).

23 Parsons, H. A. *et al.* Sensitive detection of minimal residual disease in patients treated for early-stage breast cancer. *Clinical Cancer Research* **26**, 2556-2564 (2020).

24 McDonald, B. R. *et al.* Personalized circulating tumor DNA analysis to detect residual disease after neoadjuvant therapy in breast cancer. *Science translational medicine* **11**, eaax7392 (2019).

25 Rothé, F. *et al.* Circulating Tumor DNA in HER2-Amplified Breast Cancer: A Translational Research Substudy of the NeoALTTO Phase III TrialctDNA as a Predictive Biomarker in HER2+ Breast Cancer. *Clinical cancer research* **25**, 3581-3588 (2019).

26 Riva, F. *et al.* Patient-specific circulating tumor DNA detection during neoadjuvant chemotherapy in triple-negative breast cancer. *Clinical chemistry* **63**, 691-699 (2017).

27 Zhou, Q. *et al.* Persistence of ctDNA in Patients with Breast Cancer During Neoadjuvant Treatment Is a Significant Predictor of Poor Tumor Response. *Clinical Cancer Research* **28**, 697-707 (2022).

28 Ortolan, E. *et al.* Blood-based genomics of triple-negative breast cancer progression in patients treated with neoadjuvant chemotherapy. *ESMO open* **6**, 100086 (2021).

29 La Rocca, E. *et al.* Early stage breast cancer follow-up in real-world clinical practice: the added value of cell free circulating tumor DNA. *Journal of Cancer Research and Clinical Oncology* **148**, 1543-1550 (2022).

30 Chen, Y.-H. *et al.* Next-generation sequencing of circulating tumor DNA to predict recurrence in triple-negative breast cancer patients with residual disease after neoadjuvant chemotherapy. *NPJ breast cancer* **3**, 24 (2017).

31 Takahashi, H. *et al.* Correlation of methylated circulating tumor DNA with response to neoadjuvant chemotherapy in breast cancer patients. *Clinical breast cancer* **17**, 61-69. e63 (2017).

32 Magbanua, M. J. M. *et al.* Clinical significance and biology of circulating tumor DNA in high-risk early-stage HER2-negative breast cancer receiving neoadjuvant chemotherapy. *Cancer Cell* (2023).

33 Cailleux, F. *et al.* Circulating Tumor DNA After Neoadjuvant Chemotherapy in Breast Cancer Is Associated With Disease Relapse. *JCO Precision Oncology* **6**, e2200148 (2022).

34 Shaw, J. *et al.* Serial postoperative ctDNA monitoring of breast cancer recurrence. *Journal of Clinical Oncology* **40**, 562-562 (2022). https://doi.org/10.1200/JCO.2022.40.16_suppl.562

35 Lipsyc-Sharf, M. *et al.* Circulating tumor DNA (ctDNA) and late recurrence in high-risk, hormone receptor–positive, HER2-negative breast cancer (CHiRP). *Journal of Clinical Oncology* **40**, 103-103 (2022). https://doi.org/10.1200/JCO.2022.40.16_suppl.103
